# Effectiveness of transoral robotic surgery of the base of the tongue vs. conservative treatment for obstructive sleep apnea, a RCT, the RAPID study protocol

**DOI:** 10.1016/j.conctc.2026.101619

**Published:** 2026-02-21

**Authors:** A.G.L. Toppenberg, J. van der Maten, M. Bos, W.J. Schuiling, W.L. Lodder, L.Q. Schwandt

**Affiliations:** a Department of Otorhinolaryngology – Head and Neck Surgery, University Medical Centre Groningen, Groningen, the Netherlands; b Department of Otorhinolaryngology – Head and Neck Surgery, Frisius Medical Center, Leeuwarden, the Netherlands; c Department of Pulmonology, Frisius Medical Center, Leeuwarden, the Netherlands; d Department of Neurology, Frisius Medical Center, Leeuwarden, the Netherlands

## Abstract

**Background:**

CPAP is the gold standard treatment for obstructive sleep apnea (OSA) but suffers from poor long-term compliance. Alternatives like mandibular advancement devices (MADs) and surgery, such as transoral robotic surgery (TORS), are available, but their comparative efficacy is unclear.

**Study objective:**

This study aims to evaluate whether base of the tongue (BOT) reduction using TORS is equal or superior to CPAP and MADs in improving quality of sleep and life in patients with moderate to severe OSA eligible for surgery.

**Study design:**

A prospective randomized controlled trial.

**Population:**

Patients eligible for TORS and CPAP or MAD aged >18 years with moderate to severe OSA (Apnea Hypopnea Index >15) untreated before.

**Interventions:**

In total 50 patients, 25 in each treatment arm will be randomized to either TORS or non-surgical therapy (CPAP or MAD depending on preference of patient or physician).

**Outcome measures:**

Primary outcomes will include improvement in sleep apnea severity measured through the apnea hypopnea index (AHI) and oxygen desaturation index (ODI)4%. Secondary outcomes will assess long-term quality of life compared to non-surgical therapy and adherence to devices.

**Results:**

The data will be analysed on an Intention-To-Treat principle (ITT). Ethical approval was obtained from RTPO in September of 2023. Trial registration number: NL84446.099.23 METCnumber UMCG25.327. Outcomes will be published in peer reviewed journals and presented at (inter)national conferences.

**Discussion:**

Findings of this study will address the evidence gap in the comparative effectiveness of TORS versus non-surgical therapies for OSA and may inform future clinical decision making and guideline development.

## Highlights

• There is currently insufficient evidence from RCTs regarding the efficacy of TORS compared to conservative treatment.
• To address this gap, we propose conducting a trial to evaluate whether TORS offers superior outcomes compared to conservative treatment.
• The primary endpoint is 6 month AHI, with 12 month follow up assessing durability.

## 1 Introduction

In obstructive sleep apnea (OSA) syndrome, repetitive partial or complete collapse of the upper airway during sleep leads to nocturnal hypoxia and interruption leading to daytime sleepiness [1]. OSA is associated with an increased risk of comorbidities such as hypertension, type 2 diabetes mellitus, and cardiovascular disease [2,3]. The gold standard treatment for OSA is continuous positive airway pressure (CPAP). CPAP effectively reduces apnea hypopnea index (AHI), improves sleep quality and decreases cardiovascular co-morbidities [4,5]. Regardless of the benefits, 30% of patients refuse treatment with CPAP due to several reasons including pressure effect of the mask [4] [[5], [6], [7]]. Another challenge of CPAP is treatment adherence, which requires a minimum usage of 4 h per night on at least 70% of nights. A study by Koehler et al. has shown that after 3 months adherence rates were 54% [8]. Mandibular advancement devices (MADs) are another non-surgical treatment modality that effectively reduce AHI [9], though they are less effective than CPAP. However, MADs demonstrate higher treatment adherence compared to CPAP [10].

For OSA patients who are unable to tolerate CPAP or MADs and are eligible for surgery, transoral robotic surgery (TORS) has emerged as an effective treatment option. TORS focuses on the obstruction at BOT, permanently enlarging the upper airway by removing lingual tonsil tissue. However, like all surgeries, TORS carries inherent risks such as postoperative bleeding, stenosis, infection and dysphagia, all risks that non-surgical therapies do not present. While studies have shown that TORS effectively reduces AHI [11,12], randomized trials comparing its efficacy to standard non-surgical therapies are lacking.

In this proposed study, a trial designed to address a clinically pragmatic question: whether TORS, an accepted surgical treatment for selected patients but typically positioned after non-surgical therapies in the routine care pathway, provides outcomes that are equal or superior to standard non-surgical management. In this parallel-group, randomized controlled trial with a 1:1 allocation ratio, we will compare TORS to non-surgical therapy (CPAP or MADs). Following a superiority framework, it aims to determine whether TORS provides better sleep outcomes, long-term efficacy, and quality of life while assessing complications, compliance, and functional outcomes. These comparators were chosen because CPAP and MADs are well-established, non-invasive treatments recommended in clinical guidelines and are typically chosen as first options in routine care. Historically, surgery has been considered a last resort in the management of OSA. Therefore, CPAP and MAD are pooled as non-surgical therapy in this trial to reflect real-world conventional management rather than to compare TORS against a single treatment modality. The primary objective of this study is to quantify the effectiveness of TORS relative to CPAP or MADs in treating OSA. Additionally, this study aims to determine the long-term outcomes of TORS compared to conventional therapy, assess functional sleep outcomes in patients undergoing TORS. Furthermore, it will evaluate health-related quality of life in both treatment groups and examine patient compliance with non-surgical therapy. By addressing these objectives, this study aims to provide comprehensive insight into the role of TORS as a treatment modality for OSA, helping to inform clinical decision-making and improve patient outcomes.

## 2 Methods

### 2.1 Study setting

**Fig. 1:**
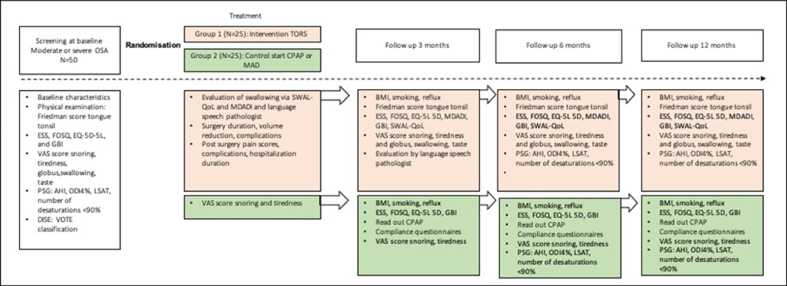
Overview of the design of the RAPID randomized controlled trial.

This study is single-site, two-arm, parallel-group, randomized controlled trial, in a general hospital, the Frisius Medical Center located in Leeuwarden, the Netherlands. The trial aims to compare the clinical effectiveness of TORS versus non-surgical therapy (CPAP or MAD) in patients with moderate to severe OSA and will include 50 patients eligible for surgery randomized into either group 1 receiving TORS BOT reduction (*n* = 25) or group 2 CPAP or MAD (*n* = 25), depending on clinical preference. The duration of the trial is estimated to be approximately two years. The trial design is visualized in Fig. 1.

### 2.2 Eligibility criteria

All patients presented at the otorhinolaryngology department of the Frisius Medical Center with a diagnosis of moderate to severe OSA eligible for surgery based on physical examination, respiratory polygraphy (PG) and drug induced sleep endoscopy (DISE) will be screened for eligibility. Inclusion criteria include [1]: age of at least 18 years or older [2]; ability to understand and comply with the protocol requirements, and provide written informed consent [3]; AHI of equal to or more than 15 on PG [4]; physical examination showing lingual tonsils classified as Friedman grade 3 or 4; and [5] DISE demonstrating collapse at BOT level classified as grade 2. Exclusion criteria include [1]: body mass index higher than 32 [2]; concentric collapse during DISE [3]; symptoms of Central Sleep Apnea Syndrome [4]; high co-morbidity as determined by American Society of Anaesthesiologists (ASA) score of 4 (severe systemic disease that is a constant threat to life) [5]; history of psychiatric hospitalization [6]; excessive use of opiates or benzodiazepines; and [7] previous treatment of CPAP and/or MAD or inability to tolerate MAD.

### 2.3 Study interventions

Participants randomized to the surgical arm will undergo TORS with BOT reduction, a technique established by Vicini et al. in 2010 [13]. In the Netherlands it is an accepted and well tolerated technique to reduce the BOT [14]. Procedures will be performed under general anaesthesia by two head and neck surgeons of the Frisius Medisch Centrum Leeuwarden, following standardized protocols. Postoperative care includes a short clinical stay of 3 days and a clinical follow-up visit at 6 weeks.

Participants randomized to the control arm will receive non-surgical therapy, consisting of the gold standard treatment, CPAP or MAD, as determined by shared decision-making between patient and their physician. CPAP devices (Airsense 10 AutoSet, Airsense 10 Elite, Airsense 11 Autoset or Airsense 11 Elite) will be prescribed by the pulmonologists of the Frisius Medisch Centrum Leeuwarden and will be tailored to patient needs. CPAP devices will be fabricated by ResMed. Adherence data will be remotely monitored.

Participants receiving MAD will be evaluated by a maxillofacial oral surgeon and fitted with custom adjustable devices manufactured by SomnoMed, SomnoDent Flex. The MAD will be made in the dental laboratory of the Frisius Medisch Centrum Leeuwarden.

CPAP and MAD users will receive standardized counseling regarding usage, side effects, and expectations. Technical or adherence issues will be addressed through telephone follow-up and in-person visits as needed.

The following interventions are prohibited during the trial period [1]: use of CPAP or MAD after intervention via TORS [2]; other concomitant transoral surgery such as (bi maxillary) osteotomy [3]; other concomitant OSA treatment such as position therapy, hypoglossal nerve stimulation, nasal resistors, oropharyngeal exercises.

### 2.4 Questionnaires

The Functional Outcomes of Sleep Questionnaire (FOSQ-35) evaluates the impact of sleep disorders on daily functioning, with higher scores reflecting better functional status [15] and is validated in Dutch. An increase of ≥2 points from baseline is considered clinically meaningful [16]. Daytime sleepiness will be assessed using the validated Epworth Sleepiness Scale (ESS) [15], which comprises eight items scored from 0 to 3, yielding a total score between 0 and 24. A score below 10 is generally considered normal among healthy adults [17]. Swallowing-related quality of life will be measured using the MDADI [18], which ranges from 20 to 100; scores between 80 and 100 suggest normal swallowing ability. The EuroQol 5-Dimension 5-Level questionnaire (EQ-5D-5L) is a standardized tool to assess health-related quality of life across five domains: mobility, self-care, usual activities, pain/discomfort, and anxiety/depression. Each domain has five severity levels, plus a visual analogue scale from 0 to 100 to rate overall health [19]. The Swallowing Quality-of-Life Questionnaire (SWAL-QoL) is a validated tool assessing how swallowing problems affect daily life. It covers physical, emotional, and social aspects, with higher scores indicating better quality of life [20].

### 2.5 Study outcomes

The primary endpoint of this study is the mean AHI measured 6 months post-intervention. This time point was selected because it reflects the earliest clinically relevant assessment at which treatment effects are expected to be stable in both arms: after postoperative recovery following TORS and after establishment of CPAP/MAD use and adherence. The PG will be performed at baseline, 6 months and 12 months. A lower AHI reflects improved airway patency and is thus clinically meaningful in assessing treatment effectiveness between groups. Twelve month follow up is included as an important secondary endpoint to evaluate durability of treatment effects in this chronic condition. Secondary outcomes include: (1) 50% reduction of AHI at 6 months compared to baseline and or AHI below 20/h (Sher's criteria) [2,21] reduction of AHI at 12 months to <5/h will be defined as surgical cure [3,22] differences in mean ODI 4% values at 6 months and 12 months, and reduction of ODI 4% to 25% relative to baseline will be defined as clinically relevant [4,22] differences between FOSQ-35 [15] scores at 3 months, 6 months and 12 months in which an increase of 10% of the score compared to baseline on the FOSQ-35 questionnaire will be defined as clinically relevant [5,22] differences between scores of ESS at 3 months, 6 months and 12 months and a reduction on the ESS to <10 will be defined as clinically relevant [6,23] differences between scores of quality of life questionnaires EQ-5D-5L [19] at 3 months, 6 months and 12 months [7]; differences in scores on the visual analogue scales (VAS) for snoring, tiredness and globus in all patients between baseline, 3, 6 and 12 months [8]; VAS scores swallowing and taste for patients in the TORS group at baseline compared to 3 months, 6 months and 12 months.

Other study parameters will be descriptive parameters such as age, gender, body mass index, history of smoking, reflux complaints, dentition, history of medication use including sleep medication, physical examination (Friedman score of the tongue tonsils and throat tonsils), measurements of DISE such as tongue base collapse classified by velum oropharynx tongue base epiglottis classification system (VOTE) [24]. Other PSG measurements are also included as study parameters such as ODI 4%, lowest desaturation (LSAT), desaturations below 90%, number of hypopneas, number of apneas and snoring time. For CPAP and MAD compliance will be assessed by pulmonologists and maxilla oral facial surgeons including follow up time, start date of treatment. Quality of life assessments and swallowing quality through MDADI [18] and SWAL-QoL [20] at baseline 3 months, 6 months and 12 months for the TORS group.

### 2.6 Recruitment and screening

In the Frisius Medical Center Leeuwarden, the routine medical care for patients suspected of having sleep apnea is as follows: patients are referred to the Multidisciplinary Sleep Centre (MDSC) by the pulmonologist, ENT surgeon, neurologist or general practitioner. All patients are discussed during the MDSC meeting after the diagnostic work-up. The diagnostic evaluation of OSA consists of multiple tests performed according to the national OSA guidelines [14]. These include completing questionnaires like the Holland Sleep Disorders Questionnaire (HSDQ) [25], ESS [23] and undergoing a PG. Depending on the results of the PG, patients may be referred to the neurologist, to the pulmonologist or ENT surgeon. In cases of suspected obstructive sleep apnea, patients are referred to the ENT surgeon for further evaluation, including physical examination and DISE. Patients demonstrating concentric collapse or no collapse of relevant anatomical structures (velum, oropharynx, tongue base or epiglottis) are considered ineligible for surgery. The findings of the examination are discussed with the patient and together with the physician a treatment plan is made that may consist of surgery via TORS (if DISE results permit), CPAP or MAD. If the patient chooses CPAP, referral to pulmonologist is indicated and if MAD is chosen, referral to maxillofacial oral surgeon is indicated. If the patient chooses surgical treatment, patient will be scheduled for surgery. After discussion of the DISE findings and available treatment options, patients iwho are eligible for both treatment arms will be informed about the clinical trial. If the patient is interested, the investigator will contact the patient for further explanation of the study.

After inclusion and randomization, group 1 will undergo TORS and will be hospitalized post-surgery, which is expected to be 3 days. Patient will be discharged home with pain medication and will visit the outpatient clinic after 6 weeks for evaluation of surgical wounds. Group 2 will be assessed by either a pulmonologist or a MFO surgeon. They will have an evaluation of treatment after 3 months. Time point 0 (T0) starts when patient undergoes surgery and the first day of usage of CPAP or MAD. The TORS group will complete questionnaires at 3, 6 and 12 months and will undergo PG at 6 and 12 months for evaluation of the treatment. Then, they will also have evaluation of swallowing quality of the speech language pathologist at baseline, 2 days after surgery (while hospitalized) and 6 weeks. The CPAP or MAD group will have outpatient follow-ups at the pulmonologist or MFO surgeon at the same time points. Then, they will also complete the questionnaires (3,6 and 12 months) and will undergo PG at 6 and 12 months. The CPAP will be read out at 3,6, and 12 months. For the TORS group, in addition to standards PG and quality of life questionnaires, pain scores using VAS will be recorded. The length of hospital stay will also be monitored.

### 2.7 Informed consent

Documented informed consent will be obtained for all patients included in the study before they are registered in the study. This will be done in accordance with the national and local regulatory requirements. The informed consent procedure will conform to the ICH guidelines on Good Clinical Practice. This implies that “the written informed consent form will be signed and personally dated by the patient or by the patient's legally acceptable representative”. Patients will be given sufficient time for consideration. An independent physician will be available accordance with the requirements of the national law. It is the responsibility of the investigator to obtain signed informed consent from every patient prior to the start of any study related procedure.

### 2.8 Sample size calculation

To answer our primary objective 25 patients per arm must be included in the study. This sample size calculation is based on results of an earlier RCT where OSA surgery was compared to conventional therapy (medical management) [26]. At 6 months MacKay et al. found the means of AHI to be in group 1, the conventional group 34.5 with a standard deviation of 23.0 and in group 2, the surgery group 20.8 with a standard deviation of 18.4. We have chosen to select a smaller standard deviation compared to the literature, as retrospective analysis of our database of OSA patients treated with TORS show a mean of AHI at 3 months (n = 31) of 16.4 with a standard deviation of 12.0 and a mean of AHI at 1 year (n = 14) of 10.8 with a standard deviation of 8.48. As our inclusion and exclusion criteria are strict and like our retrospective data and we expect a similar outcome. Given the sample size, analyses will primarily focus on estimating treatment effects with 95% confidence intervals. Secondary outcomes and subgroup analyses will be considered exploratory. Group sample sizes of 21 and 21 achieve 82% power to detect a difference of 10.0 between the null hypothesis that both group means are 34.0 and the alternative hypothesis that the mean of group 2 is 24.0 with estimated group standard deviations of 10.0 and 12.0 and with a significance level (alpha) of 0.05 using a two-sided two-sample *t*-test. The detectable differences are estimated to be of clinical relevance. To correct for possible missing data, we calculated a 20% bigger sample size which results in a sample size of 25 patients per arm.

### 2.9 Randomization

Patients fulfilling the eligibility criteria will be enrolled in this study. Patients will be randomized to group 1; BOT reduction via TORS or group 2; CPAP or MAD. Blinding of patients or investigators will not be possible as interventions will make patients aware of the group they have been allocated to. Randomization is performed using sealed envelopes prepared in advance by an independent person who is not part of the research team and is not involved in participant recruitment, consent, procedures, eligibility assessment, treatment delivery or outcome assessment. The randomization list is not accessible to the research team. The list will be generated, and individual treatment assignments will be made available via sealed envelopes. Allocation will be performed by opening a sealed envelope, in strict sequential order, after the patient has provided written informed consent, to safeguard allocation concealment.

### 2.10 Statistical analysis

All statistical analyses will be conducted using IBM SPSS Statistics. The full analysis set (FAS) follows the Intention-To-Treat (ITT) principle and includes all planned subjects, except those with major protocol violations, no post-baseline data, or no study intervention.

Baseline characteristics, including age, gender, BMI, PG, ENT exam, DISE results, smoking, and reflux history, will be summarized using descriptive statistics. Quantitative variables will be presented as mean and standard deviation or as median with interquartile range or minimum/maximum, and qualitative data as frequencies and percentages, with 95% confidence intervals where applicable. Data normality will be assessed graphically and numerically. No formal statistical tests will be performed for baseline comparisons. The primary outcome (AHI at 6 months) will be analysed using independent *t*-test or Mann-Whitney *U* test depending on data distribution. Given the RCT design, adjustments for baseline differences are not foreseen. In case of missing data this will be mentioned in the results. Missing data will not be replaced. For all hypothesis testing, a two-sided p-value of less than 0.05 indicates statistical significance. Secondary outcomes will follow the same approach. For continuous variables, appropriate group comparisons (*t*-test, Mann–Whitney U, ANOVA, or Kruskal–Wallis) will be used. For categorical outcomes, Fisher's exact or Chi-square tests will be applied as applicable. Because the control arm includes two standard non-surgical modalities (CPAP and MAD), additional prespecified analyses will be performed to assess robustness. These include [1] subgroup analyses comparing TORS vs CPAP and TORS vs MAD separately (exploratory due to sample size) and [2] sensitivity analyses adjusting for control modality (CPAP vs MAD) when evaluating the primary endpoint. Treatment adherence data will be reported descriptively and considered when interpreting outcomes. Other study parameters will be analysed descriptively as outlined above.

### 2.11 Monitoring

Although no independent Data Monitoring Committee (DMC) has been established for this study due to the relatively small sample size, the low-risk nature of the interventions (surgical and non-invasive standard therapies), and the short-term primary outcomes, all serious adverse events will be assessed by the principal investigator and study team in accordance with institutional and ethical guidelines.

In accordance with Article 10.4 of the Dutch Medical Research Involving Human Subjects Act (WMO), the study will be suspended if there is reason to believe that continuation may endanger participant health or safety. The sponsor will promptly notify the accredited METC, including the reason. The study may only resume after a positive decision by the METC. Participants will be informed by the investigator. All undesirable experiences occurring during the study, whether related to the intervention or not, will be recorded if reported by the subject or observed by the study team. SAEs include death, life-threatening situations, hospitalization or its prolongation, or lasting disability. Events that did not result in these outcomes but could have, based on medical judgement, are also considered SAEs. Elective hospital admissions are not classified as SAEs. Any SAE occurring at Frisius Medical Center Leeuwarden must be reported to the principal investigator within 24 h.

## 3 Discussion

The proposed study aims to evaluate the efficacy of TORS compared with standard non-surgical therapies for OSA. By assessing both clinical effectiveness and long-term patient outcomes, this study will provide insights into the optimal OSA treatment strategy and evaluate whether surgical intervention should be positioned earlier in the treatment pathyway or remain a secondary option in clinical guidelines. Given the chronic nature of OSA, 12-month outcomes will be used to evaluate durability of treatment effects and to support interpretation beyond the primary 6-month endpoint.

A limitation of this pragmatic study design is that the control arm includes two distinct conservative modalities (CPAP and MAD), which may introduce within-arm heterogeneity. Therefore, subgroup and sensitivity analyses by control modality are planned to assess the consistency of the primary findings. Another limitation of this trial is the relatively small sample size, which may reduce statistical power to detect modest differences between groups, particularly for secondary outcomes and repeated measures analyses, increasing the risk of type II error. Therefore, results will be interpreted with emphasis on effect estimates and confidence intervals rather than statistical significance alone, and non-significant findings will be interpreted cautiously.

Importantly, outcomes of upper airway surgery depend on careful patient selection and individualized treatment planning. DISE can support this process by identifying the relevant anatomical levels and patters of collapse, enabling more targeted surgical decision-making and potentially optimizing outcomes [27,28]. In addition, while PG outcomes such as AHI remain important objective markers of disease severity, they do not fully capture treatment benefit from the patient perspective. Therefore, quality of life daytime sleepiness, and functional outcomes should be considered key endpoint when evaluating surgical versus non-surgical treatment strategies, particularly given their relevance to long-term adherence, acceptance and overall patient benefit [29].

The findings from this study will have the potential to inform clinical guidelines and therefore help patients and healthcare providers to determine the most appropriate treatment approach. By demonstrating the potential efficacy of TORS the study could expand treatment options for patients with moderate to severe OSA eligible for surgery. Furthermore, evaluating the economic impact of TORS in comparison to long-term CPAP/MAD usage will contribute to healthcare policy discussions on cost-effectiveness of treatment in OSA management.

## CRediT authorship contribution statement

**A.G.L. Toppenberg:** Writing – review & editing, Writing – original draft. **J. van der Maten:** Writing – review & editing, Supervision, Methodology. **M. Bos:** Supervision, Investigation. **W.J. Schuiling:** Writing – review & editing, Project administration. **W.L. Lodder:** Writing – review & editing, Methodology, Investigation, Conceptualization. **L.Q. Schwandt:** Writing – review & editing, Supervision, Methodology, Investigation, Funding acquisition, Conceptualization.

## Roles and responsibilities

Sponsor is in charge of providing salary of investigators. Provides data management team, coordinating center. Protocol contributors were the authors of this protocol. AGLT (collection, analysis, interpretation of data, writing of the report), JM (study design, management), MB (management), WJS (study design, management), WLL (collection, writing of the report), LQS (study design, management, submit report for publication)

## Funding

Subsidising party Medisch Specialistisch Bedrijf Vrijgevestigd Collectief Leeuwarden.

Sponsor: Frisius Medical Center, Leeuwarden.

## Declaration of competing interest

The authors declare that they have no known competing financial interests or personal relationships that could have appeared to influence the work reported in this paper.

## Data Availability

No data was used for the research described in the article.
